# Draft genome sequence of *Paenibacillus marinisediminis* LHW35^T^ isolated from marine sediment reveals genes for ammonia assimilation

**DOI:** 10.1128/mra.00229-26

**Published:** 2026-05-18

**Authors:** Jin-Hee Seo, Rae Gyeong Chun, Kyung June Yim, Geon-Hee Park, Myunglip Lee, Hae-Won Lee

**Affiliations:** 1 Department of Food Bioengineering, Jeju National University 34926, Jeju, Republic of Korea; 2 Korea Food Research Institute (KFRI) 71645, Wanju, Republic of Korea; 3 Food Tech Center (FTC), Jeju National University 34926, Jeju, Republic of Korea; 4 Research Institute of Advanced Technology, Jeju National University 34926, Jeju, Republic of Korea; California State University San Marcos 14673, San Marcos, California, USA

**Keywords:** *Paenibacillus marinisediminis*, LHW35, Gangjin Bay, ammonia assimilation

## Abstract

We present the draft genome sequence of *Paenibacillus marinisediminis* LHW35^T^ isolated from marine sediment in Gangjin Bay, Republic of Korea. The genome annotation recovered 4,755 protein-coding genes and 92 RNA genes with a guanine-cytosine (GC) content of 46.5%. Subsystem analysis identified genes for ammonia assimilation, suggesting potential role in nitrogen cycling in marine sediments.

## ANNOUNCEMENT

*Paenibacillus marinisediminis* LHW35^T^ (KACC 16317^T^ = JCM 17886^T^) is a gram-negative, non-motile, endospore-forming, rod-shaped bacterium within the family *Paenibacillaceae* (1, 2). This strain was isolated from marine sediment collected from Gangjin Bay, Jeollanam-do, Republic of Korea (34.568483 N, 126.780070 E) using a dilution-plating technique with pleuropneumonia-like organism agar (PPLOA; Difco, Korea) at 37°C, as described previously (1). For genome sequencing, the strain was revived from a lyophilized stock and cultivated on marine agar (containing 19.45 g/L NaCl; MBcell, Republic of Korea) using the streak plate method and incubated at 30°C for 5 days. The obtained colonies were subcultured three times to achieve a pure culture and a colony obtained from the third subculture.

Genomic DNA of *Paenibacillus marinisediminis* LHW35^T^ was extracted and purified using the Qiagen MagAttract HMW DNA Kit (Qiagen, Hilden, Germany) according to the manufacturer’s protocol. The sequencing library was prepared with the TruSeq DNA Library LT Kit (Illumina, San Diego, USA) following the manufacturer’s instructions. Whole-genome sequencing was carried out on the Illumina NovaSeq platform (3), generating paired-end reads of 2 × 150 bp. A total of 7,180,913 reads comprising 2.16 × 10⁹ bases were obtained. The average read lengths for forward and reverse reads were 150 bp, with mean quality scores of 35.56 and 35.22, respectively. The data set exhibited a guanine-cytosine (GC) content of 48.87%, and 91.0% (Q30) of bases met the quality threshold. Sequence reads were processed using Shovill Galaxy version 1.1.0 (4), where trimming and assembly were performed by Trimmomatic v0.39 (5) and SPAdes v3.14.3 (6), respectively, within the Shovill environment. Default parameters were applied for all software unless otherwise specified. The assembly environment was run under the Galaxy framework, which integrates quality control, trimming, and *de novo* assembly steps in a reproducible workflow.

**Fig 1:**
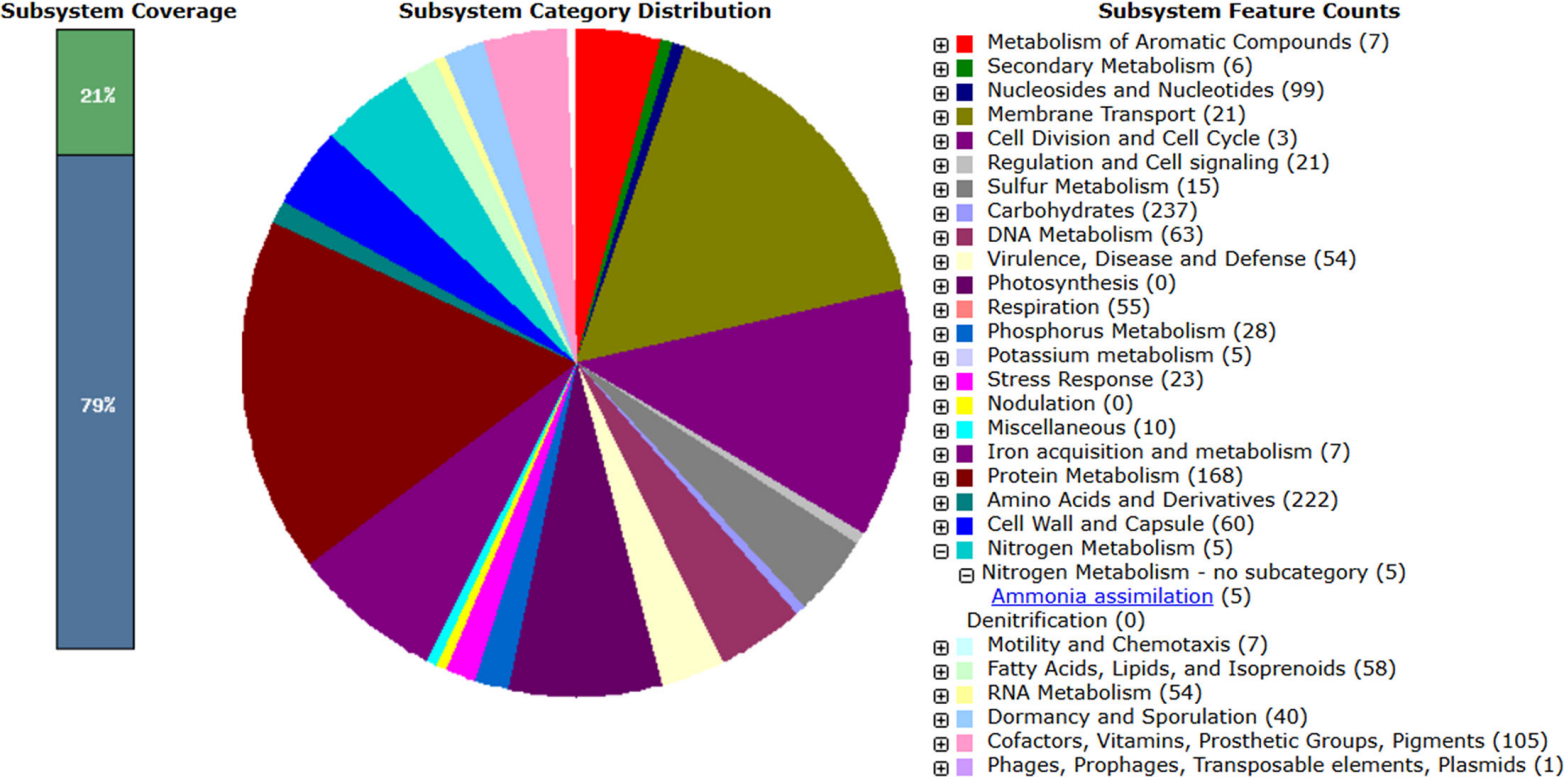
Subsystem-based functional classification of the draft genome of *Paenibacillus marinisediminis* strain LHW35 using RAST annotation.

As analyzed using the RAST server with SEED viewer, the genome of *Paenibacillus marinisediminis* LHW35^T^, which is 5,082,162 bp in size and assembled into 65 contigs according to the GenBank Assembly record, exhibits a guanine-cytosine content of 46.5%, encompasses 297 subsystems, encodes 4,755 protein-coding sequences, and contains 92 RNA genes, with an N50 value of 369,200 bp. The subsystem coverage accounted for 21% of the annotated genes. The most prominent functional categories in the genome were related to carbohydrates (237 genes), amino acids and derivatives (222 genes), and protein metabolism (168 genes), each representing major metabolic capacities (Fig. 1). Detailed subsystem analysis revealed a significant nitrogen metabolism pathway associated with ammonia assimilation (five genes).

Ammonia assimilation plays an important role in the nitrogen cycling of marine sediments by assimilating ammonium into organic nitrogen forms, thereby reducing nitrogen loss and maintaining the balance of nitrogen cycling and the sustainability of microbial metabolism within the sediments (7). Consequently, the draft genome of *Paenibacillus marinisediminis* LHW35^T^ presented in this study provides valuable insights into the microbial processes involved in nitrogen cycling along the southern coast of Korea.

## Data Availability

This whole genome project has been deposited in DDBJ/EMBL/GenBank under accession number JBTGUH000000000.1. Reads are available at the Sequence Read Archive (SRA) under accession number SRR35963196, BioProject number PRJNA1358048, and BioSample number SAMN53099241. RAST annotation files are available online at Figshare (https://doi.org/10.6084/m9.figshare.31330225).

## References

[1] Lee H-W, Roh SW, Yim KJ, Shin N-R, Lee J, Whon TW, Kim JY, Hyun D-W, Kim D, Bae J-W. 2013. Paenibacillus marinisediminis sp. nov., a bacterium isolated from marine sediment. J Microbiol 51:312–317. doi:10.1007/s12275-013-3198-2 23812811

[2] Priest FG. 2009. Paenibacillus, p 269–295. In Bergey’s manual of systematic bacteriology. Springer, New York, USA.

[3] Jeon SA, Park JL, Park S-J, Kim JH, Goh S-H, Han J-Y, Kim S-Y. 2021. Comparison between MGI and illumina sequencing platforms for whole genome sequencing. Genes Genom 43:713–724. doi:10.1007/s13258-021-01096-x 33864614

[4] Seemann T. 2017. Shovill: faster spades assembly of illumina reads Available from: https://github.com/tseemann/shovill

[5] Bolger AM, Lohse M, Usadel B. 2014. Trimmomatic: a flexible trimmer for illumina sequence data. Bioinformatics 30:2114–2120. doi:10.1093/bioinformatics/btu170 24695404 PMC4103590

[6] Bankevich A, Nurk S, Antipov D, Gurevich AA, Dvorkin M, Kulikov AS, Lesin VM, Nikolenko SI, Pham S, Prjibelski AD, Pyshkin AV, Sirotkin AV, Vyahhi N, Tesler G, Alekseyev MA, Pevzner PA. 2012. Spades: a new genome assembly algorithm and its applications to single-cell sequencing. J Comput Biol 19:455–477. doi:10.1089/cmb.2012.0021 22506599 PMC3342519

[7] Pajares S, Ramos R. 2019. Processes and microorganisms involved in the marine nitrogen cycle: knowledge and gaps. Front Mar Sci 6:739. doi:10.3389/fmars.2019.00739

